# Transcutaneous and transcranial electrical stimulation for enhancing military performance: an update and systematic review

**DOI:** 10.3389/fnhum.2025.1501209

**Published:** 2025-03-03

**Authors:** Onno van der Groen, Sara A. Rafique, Nick Willmot, Margaret G. Murphy, Eulalia Tisnovsky, Tad T. Brunyé

**Affiliations:** ^1^Defence Science and Technology Group, Human and Decision Sciences, Department of Defence, Edinburgh, SA, Australia; ^2^Defence Science and Technology Laboratory, Salisbury, United Kingdom; ^3^U.S. Army DEVCOM Soldier Center, Natick, MA, United States; ^4^Center for Applied Brain and Cognitive Sciences, Tufts University, Medford, MA, United States

**Keywords:** human performance, transcranial electrical stimulation, transcranial direct current stimulation, transcranial alternating current stimulation, military, peripheral nerve stimulation, vagus nerve stimulation

## Abstract

**Introduction:**

Electrical stimulation (ES), including transcranial electrical stimulation (tES) and transcutaneous vagus nerve stimulation (tVNS), has shown potential for cognitive enhancement in military contexts. Various types of ES, such as transcranial direct current stimulation (tDCS) and transcranial alternating current stimulation (tACS), modulate neuronal membrane potentials and cortical excitability, potentially improving cognitive functions relevant to military training and operations.

**Methods:**

This systematic review updates previous findings by examining studies published between 2019 and 2024 that investigated electrical stimulation effects on cognitive performance in military personnel and tasks. We focused on whether the studies addressed key questions about the generalizability of lab findings to military tasks, the frequency and intensity of adverse effects, the impact of repeated ES administration, and the ethical and regulatory considerations for its use in potentially vulnerable military populations.

**Results:**

Eleven studies met the inclusion criteria; most demonstrated overall low to some concerns, however, two of these had overall high risk of bias. While tES and tVNS showed some promise for enhancing multitasking and visual search performance, the results were mixed, with no reliable effects on vigilance tasks.

**Discussion:**

The reviewed studies highlight the need for a better understanding of ES mechanisms, optimal stimulation parameters, and individual differences in response to ES. They also highlight the importance of conducting high-powered research in military settings to evaluate the efficacy, safety, and ethical implications of ES. Future research should address the generalizability of lab-based results to real-world military tasks, monitor the frequency and intensity of adverse effects, and explore the long-term impacts of repeated administration. Furthermore, ethical and regulatory considerations are crucial for the responsible application of ES in military contexts, and a series of outstanding questions is posed to guide continuing research in this domain.

## 1 Introduction

Electrical stimulation (ES) involves administering low intensity (0.5 m–3.0 mA) electrical current (direct or alternating) to the surface of the scalp or skin via two or more electrodes. Mechanistic models of transcranial ES (tES) suggest that the applied electrical current propagates through the skull, dura mater, arachnoid and subarachnoid space to modulate cortical neuronal membrane potentials (Galan-Gadea et al., 2023; Lefaucheur and Wendling, 2019; Molaee-Ardekani et al., 2013; Nitsche et al., 2008; Nitsche and Paulus, 2000; Reato et al., 2019). There are several different types of ES, including transcranial direct current stimulation (tDCS), transcranial alternating current stimulation (tACS), transcranial random noise stimulation (tRNS), and transcutaneous vagus nerve stimulation (tVNS). tDCS applies a current, which can be excitatory (hypopolarization) or inhibitory (hyperpolarization), influencing local and distal networks of cortical and subcortical neurons (Kunze et al., 2016; Lefaucheur and Wendling, 2019). tACS is thought to influence neuronal oscillations, thereby affecting neuronal communication within the brain (Fries, 2005; Herrmann et al., 2013), and tRNS is a type of tACS that applies a frequency spectrum of alternating current likely acting on sodium channels (Chaieb et al., 2015; Schoen and Fromherz, 2008). Electrical current applied transcutaneously, for example, with auricular or cervical tVNS can affect cortical processing likely via modulation of brainstem activity, autonomic nervous system activity, and perhaps changes in cortical excitability (Capone et al., 2015). These distributed effects on neuronal activity can produce a broad range of behavioral effects in both clinical and non-clinical participants (Bestmann et al., 2015; Brunoni et al., 2012; Sun et al., 2021), including faster reaction times and/or improved accuracy on cognitive and motor tasks, and improved spatial working memory performance. Thus, ES holds potential for improving performance in military domains including aviation, training, and operations.

In a series of comprehensive reviews on enhancement research for military applications, tES is identified as a promising method for altering cognitive function in military personnel in addition to other interventions, for example, augmented reality, mindfulness training, and sleep modification (Brunyé et al., 2020; Davis and Smith, 2019; Feltman et al., 2019; Lu et al., 2022; Peltier et al., 2019). Research using tES to target the dorsolateral prefrontal cortex (DLPFC), medial temporal lobes, fusiform gyrus, and frontopolar regions have shown beneficial effects on cognitive functions ranging from vigilance and threat detection to executive function, face memory, and creative problem solving (Brunyé et al., 2017; Koizumi et al., 2020; McKinley et al., 2012). Despite these initial promising results, overarching conclusions from these reviews and others (including meta-analyses) consistently point to equivocal results across published research and a need to better understand a multitude of outstanding questions (see Table 1) (de Berker et al., 2013; Brunoni et al., 2012; Horvath et al., 2014, 2015, 2016; Imburgio and Orr, 2018; Paulus, 2014; Prehn and Flöel, 2015). These outstanding questions generally cover topics related to underlying mechanisms, experimental methodology, task-related outcomes, short-and long-term effects, adverse effects, individual differences, ethics and regulation, and generalizability of laboratory findings to military contexts and tasks.

**Table 1:** Outstanding research questions to guide continuing research and development with ES, with an emphasis on eventual military applications.

Outstanding questions
Can ES effects on lab performance generalize to realistic and complex military tasks?
What are the frequency and intensity of acute and/or long-term adverse effects?
What are the effects of repeated ES administration on tolerability, brain function and structure, and behavior?

A recent systematic review of transcranial direct current stimulation (tDCS) effects on performance enhancement in military contexts examined 34 articles published between 2008 and 2018 (Feltman et al., 2019). This review was restricted to randomized controlled experimental designs with military-age (18–50 years) healthy non-clinical samples. Most examined articles (26 of 34) reported some positive effects of tDCS on cognitive performance, including executive function (2), learning (6), creativity and cognitive flexibility (2), perception and attention (8), memory (3), and working memory (7). Based on the results of the review, the authors suggest promise for tES, and tDCS in particular, imparting positive effects on cognitive functions with applicability to military contexts.

The present systematic review was conducted to update the most recent review (Feltman et al., 2019). We identified articles using military personnel and/or military outcome tasks published in 2019–2024. We assessed whether the identified studies adequately addressed any of the questions posed in Table 1. We first briefly summarize the prospective application of ES in military training and operations, and some of the challenges in realizing this goal. We then discuss the questions posed in Table 1 and detail the methodology and results of our systematic review.

## 2 Cognitive performance in military contexts

Cognitive performance is a critical factor responsible for successes and failures during military training and operations with cognitive decrements estimated to account for the majority (80–85%) of accidents during military training and operations (Thomas and Russo, 2007). Many core cognitive functions are therefore foundational to the successful performance of a broad range of military tasks. The cognitive tasks demanded of military personnel vary widely as a function of military occupational specialization and level of responsibility introduced by ascending rank (echelon). According to an international expert consensus panel, critical among those cognitive functions are attention and vigilance, processing speed, cognitive control (performance monitoring, response selection, inhibition, goal selection/updating/maintenance), shifting, self-knowledge, visual perception, and understanding others’ mental states (Albertella et al., 2023). Examples of tactical-level military tasks critically involving each of these cognitive functions are detailed in Table 2.

**Table 2:** Core cognitive functions involved in successful military task performance (in descending order of importance), and example tactical military tasks engaging those core functions.

Cognitive function	Example tactical military tasks
Attention and vigilance	Surveillance and reconnaissance; crowd control operations; command and control
Processing speed	Engage targets with a weapon; correct weapon malfunctions; return fire on enemy
Cognitive control	Practice noise, light, and litter discipline; engage targets (and not non-targets) with a weapon
Shifting	Control multiple semi-autonomous assets; lead members of a team; prioritize tasks
Visual perception	Engage targets with a weapon; identify cover and concealment options; identify terrain features; evaluate a casualty; perform safety checks
Understanding others’ mental states	Camouflage yourself; identify cover and concealment options; challenge persons entering your area; crowd control operations; check status of personnel
Understanding of self	Work as part of a team; know your limits; seek assistance proactively
Working memory	Compute and convert azimuths; determine direction without a compass; relay signals to others
Language	Request medical evacuation; send a situation report; report information of potential intelligence value
Declarative memory	Perform function checks on a weapon; recognize known targets; rehearse mission plans

One unique aspect of military training and operations is that they are conducted under high levels of cognitive and physical stress, energy imbalance, sleep loss, dehydration, and thermal burden (Adler et al., 2004; Brunyé et al., 2021; Campbell and Nobel, 2009). Many of these states independently and interactively produce acute impairments of cognitive function (Brunyé et al., 2021; Flood and Keegan, 2022; Lieberman et al., 2002, 2005, 2006, 2009; Orasanu and Backer, 1996; Vartanian et al., 2018). For example, the psychological stress imposed during combat-like training of elite military units is associated with impairments of attention and vigilance, memory, and reasoning (Lieberman et al., 2005). Sleep loss slows processing speed and lengthens reaction times, lowers task accuracy, and negatively influences moral decision making (Good et al., 2020; Petrofsky et al., 2022). Calorie deprivation causes decrements in executive function (Giles et al., 2019) (but also see (Lieberman et al., 2008)); dehydration impairs executive function, attention, and motor skills (Wittbrodt and Millard-Stafford, 2018); and both cold and heat stress negatively influence higher-level cognitive functions (Martin et al., 2019).

Given that ES may hold potential for improving performance in each of these cognitive functions, it is explored as a tool to remediate cognitive decrements induced by the physical and cognitive demands of military training and operations. As such, some studies using ES interventions examine effects under conditions of relative stress and adversity, complementing basic research done in relatively comfortable settings.

## 3 Outstanding questions for research and application

In Table 1, we posed a series of questions valuable for guiding continuing research examining the prospective application of ES to military contexts and tasks. We briefly summarize each question below, and then detail the methods and results of our systematic review. A more exhaustive list of outstanding questions is included in the Discussion section, broadly motivating continuing research and application.

### 3.1 Question 1: can ES effects on performance in laboratory contexts generalize to realistic and complex military tasks?

The generalizability of human sciences, particularly in the domain of human performance, offers both opportunities and challenges when transitioning from basic research to applied military settings (Blacker et al., 2019; Goodwin et al., 2018; Hedrick et al., 1993; Shenberger-Trujillo and Kurinec, 2016). Basic research provides a foundational and mechanistic understanding of human behavior, cognition, and performance, which can inform application to military contexts such as training and operations. The perceptual, cognitive, and affective processes responsible for executing laboratory tasks are foundational, theoretically underlying the performance of any cognitive task, in any context. Basic research therefore enables the development of broadly applicable strategies for adopting new tools and technologies.

However, challenges arise in transferring discoveries made in basic research to diverse real-world scenarios. With respect to military applications, there are inherent contextual differences between controlled laboratory environments and complex military operations, inter-and intra-individual variability in human performance, operational constraints such as high-stress environments, and security concerns regarding the application of susceptible technologies to potentially vulnerable populations of military personnel (Blacker et al., 2019; Hedrick et al., 1993; Niemeyer, 2009). It is important to conduct high-powered research in military settings, with military personnel, using military tasks and relevant performance outcomes. Addressing these challenges necessitates interdisciplinary collaboration among researchers, military professionals, and policymakers to ensure that insights from basic research are effectively translated into practical applications while considering the unique complexities of military training and operations.

For example, while tDCS targeting the DLPFC shows promise for improving outcomes on abstract working memory tasks performed in laboratory settings, does it improve outcomes in relatively demanding and dynamic contexts with challenging and highly applied tasks (e.g., processing and manipulating verbal and spatial information in the context of tactical communications)? The relatively small effect sizes seen on the aggregate when examining links between tES and cognitive performance (Brunoni and Vanderhasselt, 2014; Hill et al., 2016; Horvath et al., 2015) could suggest it is unlikely to affect performance on relatively variable tasks performed in noisy contexts. Similarly, the promising effects of combining tES with working memory training may or may not transfer to similar tasks performed outside of a laboratory context. It has indeed been challenging to find evidence for such transfer within the laboratory itself (Brunoni and Vanderhasselt, 2014; Melby-Lervåg et al., 2016; Pergher et al., 2022).

### 3.2 Question 2: what are the frequency and intensity of acute and/or long-term adverse effects?

Acute adverse effects of ES administration include those occurring during or immediately after stimulation (Antal et al., 2017). An early systematic review of tDCS-associated adverse effects (Brunoni et al., 2011) found the most frequently reported effects compared to sham to be itching (39.3% vs. 32.9%), tingling (22.2% vs. 18.3%), a burning sensation at the electrode site(s) (8.7% vs. 10%), headache (14.8% vs. 16.2%), and discomfort (10.4% vs. 13.4%). However, the reporting of adverse events was generally inadequate and likely biased, limiting the ability to effectively assess their frequency, intensity, and presence across experimental conditions. A more recent review found that most adverse effects of tDCS are mild, not considered serious, and short-lived, but that relatively prolonged adverse effects can also occur—namely skin lesions; and mania or hypomania primarily in patients with depression. Similarly, these adverse events were inconsistently reported and the authors suggest that further investigations are needed to characterize their type, frequency, intensity, and duration (Matsumoto and Ugawa, 2017).

A systematic review of tVNS found the most common adverse effects to be local skin irritation from electrode placement (18.2%), headache (3.6%), and nasopharyngitis (1.7%), with a minority (2.6%) dropping out of the studies due to tolerability. Stimulation was not accounted for in the heterogeneity of effects from these studies as many of the studies did not report all parameters (Redgrave et al., 2018). A more recent systematic review and meta-analysis of auricular tVNS reported that half of the studies did not disclose whether adverse effects were recorded. The most frequently reported adverse effects were ear pain, headache, and tingling. Overall, there were no differences in the risk of adverse effects following auricular tVNS when compared to controls. There appears to be no causal relationship between taVNS and severe adverse events (Kim et al., 2022).

### 3.3 Question 3: what are the effects of repeated ES administration on tolerability, brain structure and function, and behavior?

A large systematic review and meta-analysis on tolerability found that higher levels of tDCS exposure through repeated administration (typically separated by 1 day) do not increase the incidence or intensity of adverse events, and did not vary across clinical and non-clinical groups (Nikolin et al., 2018, 2019). An additional study examining five tDCS sessions within a 25-h period found no serious adverse events, but did report mild adverse effects including scalp erythema, tingling and burning sensation at the electrode site, and a transient metallic taste (Zappasodi et al., 2018).

The effect of repeated tDCS assessed with neuroimaging has yielded variable results. No metabolite changes are observed during magnetic resonance spectroscopy following five tDCS sessions within 25-h periods (Zappasodi et al., 2018). Similarly, no change in blood-based metabolic biomarkers indicative of neuronal atrophy are observed following five tDCS sessions (Kortteenniemi et al., 2020). In contrast, three sessions of prefrontal tDCS were found to increase resting cerebral profusion in the locus coeruleus that persisted across sessions of active stimulation (Sherwood et al., 2021). Another study examining three sessions of prefrontal tDCS showed highly variable effects on resting-state functional connectivity that resulted in extremely low intra-participant reliability. Interestingly, intra-participant reliability was relatively high in a sham condition, suggesting that tDCS exerts markedly different functional effects across sessions, i.e., dose dependent effects (Wörsching et al., 2017). Moreover, low intra-individual variability is observed in tDCS-induced motor evoked potentials over the course of three sessions, suggesting a general lack of habituation (Ammann et al., 2017). These studies suggest that effects of repeated tDCS administration are very difficult to predict within and across individuals.

While preliminary evidence suggests that repeated tDCS administration is safe and tolerable, this is far from exhaustive, and more research is needed to understand how higher exposures (intensity, duration, frequency) and other types of ES affect tolerability, brain, and behavior. Much remains unknown about the chronic risk profile of ES. With the proliferation of ES devices onto the open consumer market, this is a particularly important question to consider. While laboratory studies with humans might typically consider the effects of 3–5 sessions, home users of do-it-yourself consumer devices can administer ES multiple times a day for months or years, resulting in over 100 sessions of self-administered ES (Jwa, 2015; Wexler, 2016, 2018; Wexler and Reiner, 2017, 2019). In addition to skin lesions and burns reported by home users, there are potential long-term effects of repeated exposures to direct ES of the scalp, skull, meninges, and cortex. Additional insights can be gathered from clinical trials involving multi-session tDCS administration over the course of days and weeks. For example, when examining patients with bipolar depression, Sampai-Junior and colleagues demonstrated no difference in rates of adverse events between sham and active groups after 12 daily sessions over 6 weeks (at 2 mA for 30 min), but noted some evidence for increased reports of localized skin redness in the active group (Sampaio-Junior et al., 2018). Similar findings were noted in a clinical trial examining the effect of repeated tDCS (21 sessions, 2 mA for 30 min) in patients with major depressive disorder (MDD), demonstrating no differences between groups in the frequency or severity of adverse events, while noting increased rates of local skin redness and heat or burning sensations in the active group (Borrione et al., 2024). While these results are compelling, it will be important to understand not only subject experiences but also potential effects on biomarkers of neuronal integrity over the course of dozens or hundreds of sessions.

## 4 Systematic review method

**Figure 1:**
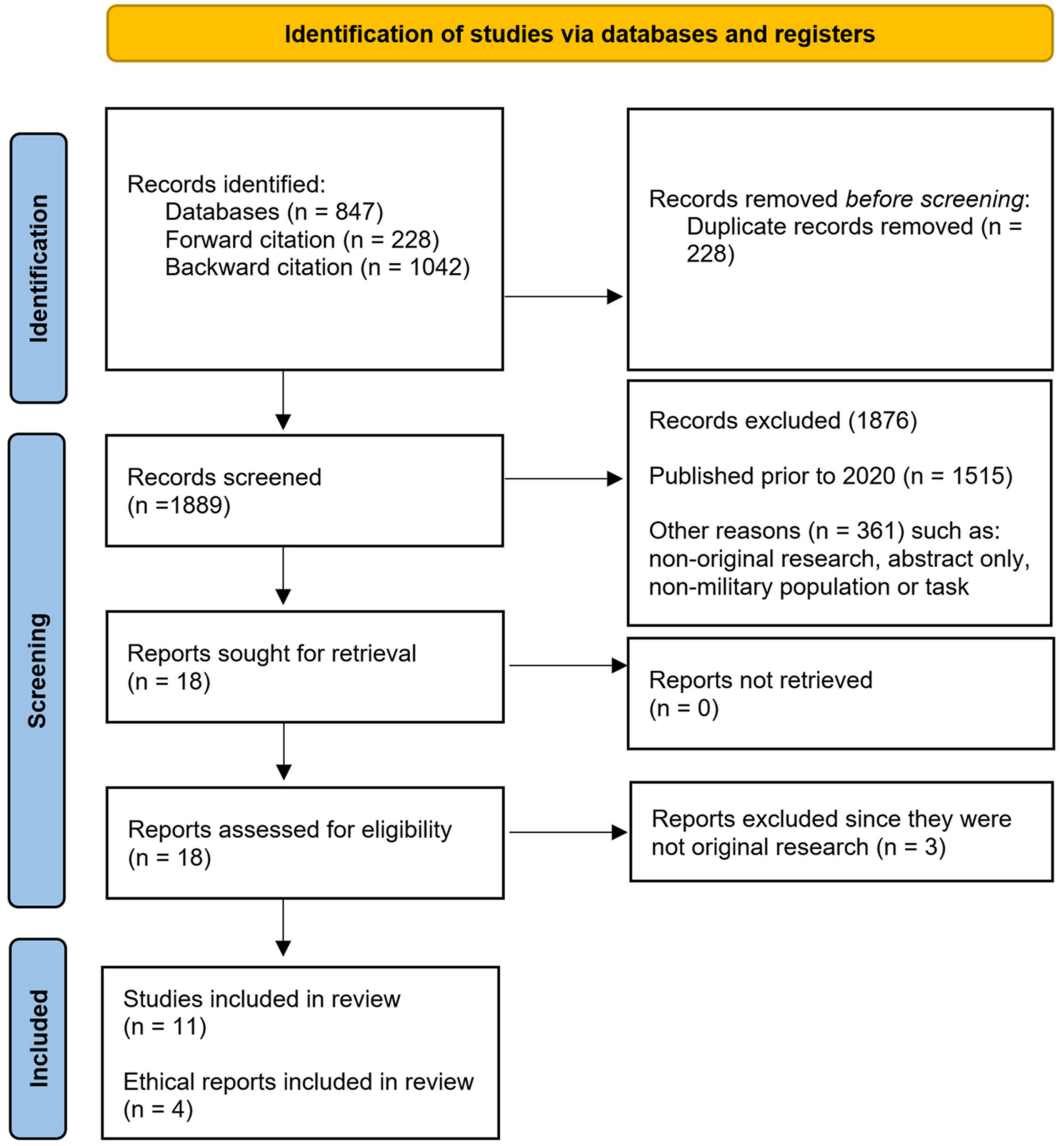
PRISMA flow diagram depicting the flow of information through the three phases of our systematic review (identification, screening, inclusion).

Herein we describe our search strategy, inclusion and exclusion criteria, study selection, data extraction, and risk of bias assessments. The full PRISMA diagram can be found in Figure 1.

### 4.1 Search strategy

Electronic searches of titles, abstracts, and keywords were conducted using the databases Scopus, Medeline, and Embase, using Boolean operators. The search terms are provided in the Supplementary materials. Database searches were conducted on December 14, 2023. No date restrictions were placed on the literature search at this point to capture forward and backwards citations (snowballing) within our timeframe. The reference lists of the included studies were screened (backward citation) as well as studies citing the included studies (forward citation) using the online tool Citation Chaser (Haddaway et al., 2022). Studies not indexed via Citation Chaser were screened manually. The results were imported in EndNote and Excel.

### 4.2 Inclusion and exclusion criteria

Full text journal articles were included if they used a non-invasive electrical brain stimulation technique (e.g., tDCS or peripheral nerve stimulation; see the Supplementary materials), focused on cognitive performance modulation, and included a military population or a military relevant task (see Table 2 for example military tasks). Studies were excluded if the abstract and title were in a language other than English, and if they were of a non-experimental nature, for example, reviews.

### 4.3 Study selection

One author (OvdG) initially screened titles and abstracts. Consequently, three authors (OvdG, TB, and SR) independently screened full text reports of all identified eligible records. Articles not in English were translated. Discrepancies were resolved through discussion and consensus. The screening process was carried out using EndNote and Microsoft Excel.

### 4.4 Data extraction

A standardized form based on the Cochrane data collection form was used for data extraction of relevant study characteristics, including general information, and study eligibility screen. If the study was found to be eligible, additional data were extracted from the methods, results/outcomes, and discussion (e.g., strengths and limitations) sections.

### 4.5 Risk of bias assessment

The Cochrane risk of bias assessment tool, RoB2, was used to evaluate the methodological quality of each included study (Sterne et al., 2019). This tool included biases induced due to the randomization process, deviation from the intended intervention, missing outcome data, outcome measures used, and bias in selection of presented results. The risk bias for each study was categorized as ‘low risk, ‘some concerns’ or ‘high risk’ automatically by the algorithm, and then again by a consensus meeting between three assessors (authors TTB, MGM, ET). Table 3 reports the results of this process for both the algorithm and assessors.

**Table 3:** Characteristics of included tDCS studies.

Reference, risk of bias (algorithm | assessors)	Design, groups, N (group)	Population (healthy), total N, N (males)|*N* (females)	Stimulation Modality, target area, electrode positioning	Stimulation intensity, density, anode and cathode size, duration	tDCS timing	Outcome task(s)	Outcome summary
Blacker et al. (2020) ROB: High | Some Concerns	Between-subjects, 2 groups (active, sham), 36 (active) 36 (sham)	Mixed population civilian and military (mean age: 26.38 years), *N* = 74, 58 M|16 F	tDCS Anode at right PPC (P4), return at contralateral R bicep.	2 mA 0.199 mA/cm^2^ 1.6 cm diameter of electrode 30 min (active) 30 s (sham)	Online	Object recognition task, search strategy training task.	Overall: No effects.
Dai et al. (2022) ROB: Some Concerns|Some Concerns	Between-subjects, 2 groups (active, sham), 15 (active), 14 (sham)	Participants from Air Force Medical University (mean age: 21.15 years) *N* = 29, 29 M | 0 F	tDCS Anode at left dlPFC (F3) and return at right supraorbital (Fp2)	1.5 mA 0.06 mA/cm^2^ 25 cm^2^ 30 min (active) 30 s (sham)	Offline	EEG, Mackworth Clock Task, Oddball task, Go/NoGo task.	Overall: Neural but not behavioral effects. Mackworth Clock Task: no effects of stimulation condition; increased P2 amplitude in active vs. sham. Go/NoGo task: no effects of stimulation condition; increased N2 amplitude in active vs. sham. Oddball task: No behavioral results.
Fatideh et al. (2023) ROB: High | High	Within-subjects, 3 conditions (left anodal, left cathodal, sham)	Military aviators (mean age: 37.53 years) *N* = 12 12 M | 0 F	tDCS Anode at right dlPFC (F4), return at left dlPFC (F3)	2 mA 0.08 mA/cm^2^ 20 min (active) 30 s (sham)	Offline	Visual Search Task, Continuous Performance Task.	Overall: Beneficial. Visual search task: Faster RT and higher accuracy in anodal versus cathodal or sham. Continuous performance task: higher accuracy in anodal versus cathodal or sham.
Kim et al. (2021) ROB: Some Concerns | Some Concerns	Between-subjects, 3 groups (1 mA, 2 mA, sham) 13 (active 1 mA) 16 (active 2 mA) 22 (sham)	Active-duty AF military (mean age: 28 years), *N* = 51 (9 dropped-out) 38 M | 13 F	tDCS Anode at left dlPFC (F3) and return at right bicep	2 mA stim: 0.199 mA/ cm^2^ 1 mA stim: 0.99 mA/cm^2^ 30 min (active) 30 s (sham)	Online	fMRI, Mackworth Clock Test.	Overall: Neural but not behavioral effects. fMRI: Increased connectivity from BA 9 to FPCN. Mackworth Clock Test: Results not reported.
McIntire et al. (2021) ROB: Some Concerns | Low	Between-subjects 2 groups (active, sham) 20 (active) 20 (sham)	Active-duty AF (mean age: 26 years) *N* = 42 (2 dropped-out) 33 M|7 F	Cervical VNS Electrodes over L or R cervical vagal nerve/neck.	Unspecified mA (varied inter-participant), 25 Hz Stimulated for total of 4 min each side (left, right)	Offline	Mackworth Clock Test, PVT, N-back Task, MATB Task, Subjective Mood Questionnaire.	Overall: Mixed. Mackworth clock task: No effects. N-back task: No effects. PVT: Higher a’ performance in active versus sham condition. MATB task: Higher throughput in active versus sham condition. Subjective: Lower fatigue and greater energy in active versus sham condition.
Nelson et al. (2019) ROB: Some Concerns | Some Concerns	Between-subjects, 2 groups (active, sham) 8 (active) 8 (sham)	Active-duty military participants *N* = 20 (16 analyzed) 16 M | 4 F	tDCS Anode over left dlPFC, return over right bicep.	2 mA 0.199 mA/cm^2^ 30 min	Online	MATB Task	Overall: Beneficial. MATB task: Higher throughput with active versus sham tDCS.
Sherwood et al. (2021) ROB: High | High	Between-subjects 3 groups (1 mA, 2 mA, sham) 15 (sham) 15 (1 mA) 17 (2 mA)	Participants recruited from Wright AF Base (mean age: 27.9 years) *N* = 47 38 M | 9 F	tDCS Anode over left dlPFC (approx. F3), return over contralateral bicep.	30 min (1 mA) 30 min (2 mA) 30s of 2 mA + 29.5 min of no stim (sham)	Online	fMRI for Resting CBF, Mackworth Clock Test	Overall: Neural but not behavioral effects. fMRI: Reduced resting CBF in active versus sham conditions. Mackworth Clock Task: No results reported.
Smits et al. (2023) ROB: Low | Low	Between-subjects, 2 groups (active, sham) 37 (active) 35 (sham)	Military service members *N* = 74 (62–72 analyzed) 68 M | 4 F	tDCS Anode over right dlPFC, return over C2	2 mA, 0.22 mA/cm^2^ (anode), 0.057 mA/cm^2^ (cathode) 20 min	Online	Threat of Shock Task, Emotional Working Memory Task	Overall: Mixed. Threat of shock responses: No effects. Working memory task: No effects. Exploratory analyses suggest baseline theta/beta ratio predictive of tDCS effects on working memory.
Wang et al. (2022) ROB: Some Concerns | Some Concerns	Between-subjects, 4 groups (ICT + tVNS, ICT + sham tVNS, sham ICT + tVNS, and sham ICT + sham tVNS) 14 (ICT + tVNS) 15 (ICT + sham tVNS) 15 (sham ICT + tVNS) 14 (sham ICT + sham tVNS)	Military medical university members *N* = 60 (58 analyzed) 60 M | 0 F	Auricular VNS Stimulation was applied to the left cymba conche	Unspecified mA (varied inter-participant), pulse width of 200–300 μs at 25 Hz and a biphasic pulse interval of 30 s ON and 30 s OFF, administered for a total of 60 min.	Online	Go/NoGo and Stop-Signal	Overall: Mixed. Stop-signal task: Improved training and near transfer when tVNS is combined with ICT versus tVNS or ICT alone. Go/No-go task: Improved training and near transfer when tVNS is combined with ICT versus tVNS or ICT alone. Stroop task: No effects.
Willmot et al. (2023) ROB: Some Concerns | Low	Between-subjects, 3 groups (active rIFG, sham rIFG, active V1) 33 (active rIFG) 34 (sham rIFG) 31 (active V1)	Members of Australian Army’s Royal Australian Armoured Corps *N* = 98 92 M | 8 F	tDCS Anode over right IFG (F10), return over left mastoid. OR Anode over V1 (Oz), return over left mastoid.	2 mA, 0.08 mA/cm^2^, 25 cm^2^, 25 min	Mixed	Visual Threat Detection Task	Overall: No effects.
Zhu et al. (2023) ROB: Some Concerns | Low	Between-subjects, 4 groups (6 Hz, 10 Hz, 20Hx, and sham) *N* = 15 in each group	Undergraduate students (mean age: 23.02 years) *N* = 60, 28 M | 32 F	HD-tACS Anode at left dlPFC (F3), return electrodes at F5, F1, FC3, AF3.	1.5 mA, 0.33 mA/cm^2^, 4.52 cm^2^, 20 min	Offline	Go/NoGo Task, Stroop Task.	Overall: Mixed. Go/No-go task: No effects. Stroop Task: Faster RT and higher accuracy in active versus sham condition.

## 5 Results

A total of 15 records were included in our review, 11 of which report experimental research, and four of which review ethical challenges. For each of the 11 experimental records, Table 3 details the risk of bias, design, sample size, targeted brain area, electrode size and positioning, stimulation intensity, density, duration, timing of stimulation, outcome tasks, and a summary of outcomes. For the four ethics reviews, we include them in the Discussion section.

### 5.1 Overall methodological patterns

According to assessors’ evaluations, most studies had overall low risk (4) or some concerns (5) of bias. Two studies demonstrated overall high risk of bias.

All but three studies administered tDCS; the others administered tACS or tVNS (transauricular or cervical). The vast majority (10/11) of studies used a between-participants design with either exclusively military personnel as participants or a mixed sample (i.e., military and civilian). Overall sample sizes varied from small to large (*N* = 12–98), but were small to medium when divided into groups in between-participants designs (n = 8–34).

Studies using tDCS or tACS variably administered from 1 m to 2 mA intensity for 20 m–30 min, with most stimulating during (online, 5) or prior to (offline, 4) task performance, and two studies stimulating both prior to and during task performance (mixed). The cortical target sites were the left (6) or right (2) DLPFC, right posterior parietal cortex (PPC, 1), right inferior frontal gyrus (IFG, 1) and primary visual cortex (V1, 1) using highly varied electrode types (e.g., sponge versus sintered ring) and surface areas (and thus current densities).

Studies using tVNS variably administered stimulation to auricular or cervical vagus afferents, with a consistent stimulation frequency of 25 Hz but different durations of 8 and 60 min. In both cases, stimulation intensity was individualized based on each participant’s pain or discomfort threshold, but the authors did not disclose actual stimulation intensity. Note that in some cases, the latter may be due to inadequate disclosure of proprietary stimulation parameters by device manufacturers.

A wide range of outcome tasks were administered across studies. The most commonly employed task was the Mackworth Clock Test (4), generally employed as a measure of vigilance. Other tasks included the diffuse & focused attention tests, a continuous performance task (CPT; measuring vigilance and attention), a psychomotor vigilance task (PVT; measuring vigilance), object recognition, visual search, oddball task (measuring target detection), the Multi-Attribute Task Battery (measuring multitasking), Go/No-Go (measuring impulsivity), Stroop tasks (measuring executive control), an emotional working memory task, and a threat of shock paradigm (measuring startle responses). In Table 2 we detailed 10 core cognitive processes important for common military functions; in general, the identified studies targeted most (6) of these with the absence of outcome tasks related to understanding the self and others’ mental states, shifting, language production and comprehension, and declarative memory.

In addition to behavioral outcomes, three studies measured brain activity using electroencephalography (EEG), and two using magnetic resonance imaging (MRI). In two of those cases, behavioral results were either not measured or not reported. Specifically, Sherwood et al. (2021) and Kim et al. (2021) used MRI and administered the Mackworth Clock Test to participants but did not measure and/or report behavioral data from this task. Dai and colleagues (Dai et al., 2022) used EEG and administered the Mackworth Clock Test and Go/No-Go task, and did report behavioral results.

### 5.2 Overall results patterns

For the tDCS or tACS studies, most (5) reported no effects of stimulation on any measured behavioral outcomes, or did not report behavioral outcomes. This included studies targeting the left DLPFC (Dai et al., 2022; Kim et al., 2021; Sherwood et al., 2021), the right PPC (Blacker et al., 2020), the right IFG, and V1 (Willmot et al., 2023). These studies measured outcomes on vigilance, executive function, visual search, and object recognition tasks.

Two of the tDCS/tACS studies showed mixed results, with stimulation targeting the left DLPFC enhancing performance on one but not another task. Zhu and colleagues (Zhu et al., 2023) reported faster reaction times and higher accuracy on a Stroop task in active versus sham tACS conditions, but no effect of stimulation condition on the Go/No-Go task. In general, the Stroop task focuses on selective attention and managing interference, more consistently engaging the anterior cingulate cortex (ACC) and DLPFC. The Go/No-Go task instead focuses on inhibiting prepotent motor responses, with a more consistent involvement of the right IFG and basal ganglia. Given that the authors targeted the left DLPFC with tACS, this may explain why positive effects were found with the Stroop but not Go/No-Go task. Smits et al. (2023) found no effect of active versus sham tDCS on startle response or working memory task performance; however, exploratory analyses did suggest that baseline EEG theta:beta ratio might predict whether anodal versus sham stimulation affects working memory performance.

Finally, the two remaining tDCS studies showed beneficial results of tDCS on visual search, vigilance, and multitasking tasks. First, Nelson et al. (2019) reported higher throughput on the MATB task in the active versus sham group with online anodal stimulation (2 mA) targeting the left DLPFC. This study did however employ a small sample size (*n* = 8 per group) which reduces the statistical power, leading to unreliable results and increasing the risk of both Type I (false positive) and Type II (false negative) errors. Studies with small sample sizes also make findings less representative of the broader population, limiting the generalizability and reproducibility of the results. Second, Fatideh et al. (2023) administered 2 mA of offline tDCS to the right DLPFC, and reported faster reaction times and higher accuracy in the anodal versus cathodal or sham conditions on both visual search and vigilance tasks. However, this study also employed a small sample size (*N* = 12), and demonstrated overall high risk of bias. The results of these studies should therefore be interpreted with caution.

The two studies using tVNS showed mixed results. McIntire et al. (2021) administered either active or sham cervical tVNS offline before participants completed the Mackworth Clock Test, N-back task, PVT, and MATB task. The authors reported higher throughput on the MATB task, and higher discriminability on the PVT in the active versus sham group; however, no effects were found on the Mackworth Clock Test or N-back test. Wang et al. (2022) combined inhibitory control training with simultaneous (online) active or sham auricular tVNS, and measured outcomes on a Stop-Signal task, Go/No-Go task, and Stroop task. The authors found beneficial effects of active versus sham tVNS on the Stop-Signal and Go/No-Go tasks (accelerated training and near transfer effects), but no effects on the Stroop task. The discrepancy in results in these two tVNS studies are likely due to the use of different stimulation sites (i.e., auricular versus cervical), stimulation durations (i.e., 8 versus 60 min), or other experimental factors. It may also be the case that tVNS can benefit vigilance, multitasking, Stop-Signal, and Go/No-Go task performance by enhancing arousal, attention, and response inhibition through its putative effects on the autonomic nervous system and the locus coeruleus-norepinephrine (LC-NE) system. However, it may not significantly benefit working memory or Stroop task performance because these tasks rely on specific executive functions and neural circuits (such as the DLPFC and ACC) that may not be as directly influenced by the generalized autonomic nervous system effects of tVNS. These remain open questions for continuing research.

## 6 Discussion

We began this review by posing a series of three outstanding questions for researchers and practitioners interested in the potential for ES methods to enhance performance in military training or operations. The first question was whether ES effects seen with validated laboratory tasks can generalize to relatively realistic and complex military tasks. Across the included studies, most cognitive tasks were validated and commonly used laboratory tasks, including the Go/No-Go, Stroop, CPT, startle response, and working memory tasks. However, relatively applied and military relevant tasks were also used in some studies; these included the Mackworth Clock Test, the MATB, and visual search and threat detection tasks.

The studies identified in this review found no evidence that performance on the Mackworth Clock Test is modulated with tDCS or tVNS. This is a somewhat surprising finding given prior research demonstrating effects of similar tDCS parameters on this task (McIntire et al., 2014, 2017). Evidence that MATB performance can be modulated was positive, with one tDCS study suggesting performance improvement with tDCS (Nelson et al., 2019), and one study suggesting improvements with tVNS (McIntire et al., 2021). For visual search and threat detection tasks, results were mixed. For example, in a within-subjects study, Fatideh and colleagues applied 2 mA bilateral tDCS to the DLPFC (F_3_, F_4_) of Iranian aviators (*N* = 12) over 3 sessions separated by 48 h (Fatideh et al., 2023). Their findings suggested that visual search improved with right anodal DLPFC stimulation, relative to left DLPFC anodal or sham stimulation. In contrast, in a repeated measures (2 × 2) study featuring a larger sample (*N* = 74; *n* = 18, 19, 18, 17) of US military personnel, participants received anodal or sham tDCS to the right PPC as they underwent training in one of two visual search tasks across three identical sessions (Blacker et al., 2020). The authors found participants improved their performance in response to both training paradigms, those being object recognition and visual search strategy, but that these improvements were unaffected by right PPC stimulation. Moreover, in a between subjects study involving Australian Army soldiers (*N* = 74), Willmot and colleagues found that participants receiving 2 mA tDCS to the right IFG or the V1 during a militarized threat detection task did not perform significantly better than those receiving sham stimulation to the rIFG (Willmot et al., 2023), contrary to previous studies using civilian samples (Clark et al., 2012). It could be the case that right PPC or IFG stimulation are less likely to modulate applied visual search and threat detection tasks, whereas right DLPFC stimulation holds more potential. However, given the large difference in sample sizes amongst the aforementioned papers, and the variable training level of the military personnel, it is a much more parsimonious explanation to reason that effects of tDCS could in fact be lessened by both the inclusion of larger samples in the later two studies (Minarik et al., 2016). In addition, there could be an interaction between prior expertise of participants and the training task, reducing the effectiveness of tDCS (Brunyé et al., 2014; Sánchez-Kuhn et al., 2018). Returning to Table 2, continuing research should also begin to examine other important and military-relevant cognitive tasks such as understanding the self and others’ mental states, language production and comprehension, and declarative memory.

The second question posed in the introduction related to the frequency and intensity of acute and/or long-term adverse effects of ES. Most studies (8/11) unfortunately did not report the presence of cutaneous sensations or adverse effects in subjects. In the three papers that did report, results were mixed. Zhu et al. (2023) found that tACS elicited “mild skin sensations” in five of the 60 participants, and none reported visual phosphenes. Smits et al. (2023) reported that one participant had a skin lesion that healed within a week, and some participants reported other mild cutaneous sensations (burning, itching, tingling); however, there were no significant differences in adverse effects between active and sham conditions. Kim et al. (2021) measured and reported cutaneous sensations, noting that there were no significant differences in adverse effects between the active and sham condition. Based on these reports, mild cutaneous sensations appear to rarely occur (in about 10% of a sample), though there is an improbable but possible risk of more serious cutaneous injury (burns, lesions). Measuring and reporting the frequency and intensity of adverse effects is critically important given that military personnel are considered a vulnerable population, with discomfort and adverse effects detracting from occupational duties, and that acceptance and adoption of novel biotechnologies might be limited by perceived discomfort. Adverse effects and drop-out rates are often used as proxy measures of acceptability/participant perspectives (van der Groen, 2023). However, intervention acceptability by the end-user and stakeholders is implicated in intervention uptake, adherence, and overall effectiveness (Sekhon et al., 2022). Continuing research should place more emphasis on measuring and reporting acceptability, and adverse events.

The third question posed in the introduction related to the effects of *repeated* ES administration on tolerability, brain function and structure, and behavior. Across the included studies, stimulation durations were predominantly limited to single sessions of 20m–30 min of tES or 8m–60 min of tVNS, which do not provide new information regarding relatively sustained or repeated stimulation conditions. There was one exception, with Fatideh et al. (2023) administering 20 min of tDCS repeatedly over three sessions. While the authors did assess participants’ mood states, they did not report information regarding cutaneous sensations or adverse effects, and did not assess effects on brain function or structure.

### 6.1 Seven outstanding questions to guide continuing research

Several additional questions are important to examine as scientists and practitioners considering adopting tES, and/or tVNS in military training or operations.

#### 6.1.1 Elucidating mechanisms at multiple scales

What are the mechanisms underlying ES effects at the micro, meso, and macro scales? Models of ES effects can be generally trifurcated into characterizing the microscopic, mesoscopic, and macroscropic levels of description (Bestmann et al., 2015). There are several putative mechanistic models of ES, mainly surrounding tDCS effects on cortical function. At the microscopic level, models tend to focus on neuronal membrane polarization induced by electrical currents. While many models and empirical research emphasizes sliding-scale models (i.e., anodal excitatory, cathodal inhibitory), more recent research suggests a much more complex and nonlinear series of functional activity changes with tDCS (Batsikadze et al., 2013; Bonaiuto and Bestmann, 2015; Molaee-Ardekani et al., 2013; Rawji et al., 2018; Silvanto et al., 2008; Vergallito et al., 2023). On the mesoscopic scale, models tend to focus on the effects on activity of relatively distributed populations and networks of cortical and subcortical neurons. Research has showcased tDCS effects on resting-state functional network connectivity in the motor network, sensorimotor network, default mode network, frontal–parietal network, and self-referential network (Chan et al., 2021; Fischer et al., 2017; Mencarelli et al., 2020; Peña-Gómez et al., 2012), including both intrinsic activity within a given network and extrinsic activity between networks. Interestingly, there is a pronounced gap between microscopic and mesoscopic models and what should be expected behaviorally from ES administration. This gap unfortunately makes it difficult or impossible to predict the behavioral consequences of ES administration, whether related to performance improvement or degradation (Bestmann et al., 2015; Bonaiuto and Bestmann, 2015).

#### 6.1.2 Isolating and characterizing parameter effects

How do stimulation parameters modulate ES effects, and what is their optimal combination? There is a complex parameter space surrounding the administration of ES, including the targeted brain region, the number and size of electrodes, the polarity, intensity, timing (i.e., online versus offline), duration of stimulation, and whether the stimulation is personalized to individual structural or functional brain characteristics. Each of these parameters independently influences the behavioral and functional effects of ES, which likely have yet unknown interactive effects (Ehrhardt et al., 2021; Filmer et al., 2019; Friehs and Frings, 2019; Ho et al., 2016; Jacobson et al., 2012; Kessler et al., 2013; Opitz et al., 2015; Saturnino et al., 2019; Truong et al., 2013; Živanović et al., 2021). Given the inherent flexibility of ES stimulation parameters and their variability across studies, it is difficult to predict the optimal combinations of parameters to influence cognitive function (Au et al., 2017; Khorrampanah et al., 2020; Wagner et al., 2016). This is a challenging combinatorial modeling problem, especially given that very few studies manipulate two or more parameters across multiple (i.e., >2) levels. Recent research has attempted to use hierarchical Bayesian meta-regressions to predict the magnitude of tDCS effects as a function of 10 stimulation parameters (including electrode number, polarity, intensity, timing, duration), and identify optimal parameter combinations for maximizing effects on five performance domains (motor skill acquisition, visual search, working memory, vigilance, inhibitory control) (Santander et al., 2024). The model did not successfully converge on optimal solutions to confer reliable predictions to the five behavioral outcomes, largely due to extensive variability across research studies (e.g., experimental methods, tasks, and outcome measures). A relatively domain-general outcome suggested an advantage to anodal versus cathodal stimulation, but this pattern was not found in any specific outcome domain. The authors conclude that there is an urgent need for research to parametrically manipulate and probe independent and interactive effects across relatively complex parameter spaces.

#### 6.1.3 Understanding effects of individual differences

How do individual differences influence ES effects? The effects of individual differences on ES-related outcomes have been examined in three primary ways. First, studies have examined how individual differences in brain structural and functional characteristics affect physiological and behavioral responses to ES. For example, individual differences in prefrontal cortical thickness influence tDCS effects on decision-making (Filmer et al., 2019). Additionally, tDCS-induced changes in resting-state functional connectivity are associated with differences in visual object-matching task performance (Pupíková et al., 2022). Secondly, studies have examined how individual differences in personality traits affect physiological and behavioral responses to tES (Brunyé et al., 2014, 2015; Krause and Cohen Kadosh, 2014). For example, tDCS affects reading speed of social sentences in readers with low scores on the behavioral approach and inhibition scales, but not those with high scores (Reyes et al., 2021); and trait anxiety modulates the effects of tDCS on creative task performance (Xiang et al., 2021). Third, studies have examined how individual differences in baseline knowledge or task proficiency affect physiological and behavioral responses to ES (Splittgerber et al., 2020). For example, tDCS increases the creativity of improvised instrument play for novices but harms expert performance (Rosen et al., 2016); and those with lower baseline reading proficiency show greater positive effects of tDCS on cross-language speech production (Bhattacharjee et al., 2020). Given the inherent heterogeneity of military personnel, the effects of relatively invariant individual characteristics is an important research topic. Individually tailored paradigms are likely needed, factoring in complex interacting variables (e.g., stimulating parameters, physiological, anatomical, and genetic differences). Personalized protocols do however raise challenges in a military setting, such as time constraints and practicalities. Recent research has further demonstrated large intra-individual variability in responses to repeat sessions of tDCS in that individuals did not respond consistently to tDCS when applied repeatedly over time (Willmot et al., 2024).

#### 6.1.4 Quantifying enhancement beyond baseline functioning

Can ES support and optimize performance, or does it truly enhance performance beyond baseline functioning? While the notion of cognitive enhancement is intriguing (Bostrom and Sandberg, 2009; Farah, 2015; Farah et al., 2014), it is challenging to truly demonstrate enhancement as opposed to performance sustainment or optimization, *per se* (Brunyé et al., 2020). Several philosophical positions conceptualize that enhancement must improve functioning of an individual beyond their normal range (Agar, 2013; Menuz et al., 2013). True enhancement would transcend the biological limits shaped by millennia of evolution—defining such biological limits will be critical, both within and across individuals. Demonstrating true enhancement requires quantitatively establishing baselines of an individual’s optimal biological performance potential under optimal conditions. For example, under an idealized set of hypothetical conditions, what could an individual achieve for reaction times, accuracy levels, or any other outcome of interest? Only when biotechnology-induced performance exceeds what has been established as innate optimal performance can it truly be deemed enhancement. In most cases, including when ES mitigates the performance deleterious effects of a contextual factor (e.g., sleep deprivation, stress, fatigue) (Hart-Pomerantz et al., 2024), performance is being sustained or optimized relative to a control condition that attempts to mimic some aspects of active experimental conditions (e.g., sham procedures with ES). These are important empirical comparisons, but they may not allow us to quantify the biological limits of performance or make inferences about enhancement. Moreover, military personnel might already be at their performance upper limit due to their training, limiting the effectiveness of ES. For example some tDCS studies in military samples did not observe any additional performance benefits (Blacker et al., 2020; Willmot et al., 2023). Visual search is required in many professions where an undetected threat, such as a weapon, can put the well-being of others at risk. Given the importance of detecting these threats, researchers have used various experimental techniques to improve performance in visual search tasks, albeit with varying degrees of success. Here, we explore two promising techniques to improve visual search using ecologically valid synthetic aperture radar stimuli: object recognition training and search strategy training. Search strategy training is intended to make observers search more systematically through a display, whereas object recognition training is intended to improve observers’ ability to recognize critical targets. Search strategy training was implemented by instructing participants to scan through the display in a pre-specified pattern. Object recognition training was implemented by having participants discriminate between targets and non-targets. We also manipulated whether observers received anodal or sham transcranial direct current stimulation (tDCS) during training, which has been shown to improve visual search performance and target learning. To measure the effectiveness of the training and stimulation conditions, we tested object recognition accuracy and overall visual search performance before and after three sessions of increasingly difficult training. Results indicated that object recognition training significantly improved object recognition accuracy relative to the search strategy group, whereas search strategy training was effective in improving visual search accuracy in those who adhered to the training. However, tDCS did not interact with training type, and although both training types yielded significant improvements, training-related improvements were not significantly different between the different approaches. This evidence suggests that strategy-based training could be as effective as the more prototypical object recognition training. Moreover, interactions between baseline performance and tDCS effectiveness have been demonstrated (Splittgerber et al., 2020). Whether ES can further improve performance in high performing individuals remains an open question.

#### 6.1.5 Identifying trade-offs and net zero-sum dynamics

Do trade-offs exist between targeted and untargeted performance domains? Using ES to enhance cognitive function raises questions about potential trade-offs between targeted and untargeted brain regions or tasks, aligning with the concept of net zero-sum dynamics within the nervous system. The net zero-sum framework posits that enhancements in one cognitive domain may be counterbalanced by detriments in another, necessitating careful consideration of cost–benefit interactions across various levels of neural processing (Brem et al., 2014; Luber, 2014). The concept underscores the need to assess potential costs alongside any enhancements. Multi-parameter considerations encompassing magnitude, duration, reversibility, and the level of impact across micro to macro scales are essential for delineating cost–benefit relationships. Within this framework, the notion of processing power emerges as pivotal, representing the brain’s capacity to allocate resources dynamically across different cognitive demands. Trade-offs, reflecting competition between sub-processes independent of top-down control, may elucidate interactions among neural elements. For instance, the speed-accuracy trade-off in decision-making tasks illustrates resource allocation dynamics, where competing demands for processing power influence task performance. While identifying the cost of ES-related enhancement remains challenging, the conceptualization of net zero-sum dynamics underscores the intricate interplay between cognitive enhancements and associated costs, necessitating nuanced framing and empirical investigation. Given the inherent complexity of military training and operations, we believe these investigations are highly warranted as military personnel are increasingly expected to perform multiple complex tasks simultaneously, recruiting diverse cognitive processes. If we are able to facilitate a few of those processes, what might happen to the other untargeted processes?

#### 6.1.6 Integrating into military training and operations

How will the use of ES devices integrate into existing military training and potentially be integrated into existing battle ensembles worn by military personnel? Schedules within military training establishments are increasingly facing time constraints and the introduction of an additional training requirement would add stress to an already overburdened system. It might be that the best solution is to identify critical periods of training where insertion of ES alongside existing training can yield performance improvement, mitigate performance decrements, or facilitate accelerated recovery. Another option could be to introduce effective interventions into the ongoing physical and wellbeing activities undertaken by military personnel during normal operation periods, thereby increasing the possibility of effective acceptance and implementation. Integrating devices into military personnel’s existing individual ensemble would involve a systematic process from research and development to acquisition and integration. Initially, the readiness and feasibility of ES technology would be assessed using the technology readiness level scale, considering factors such as safety, effectiveness, and compatibility with existing equipment (Small Business Association, 2024). Research and development efforts would focus on optimizing ES devices for military use, involving the development of prototype devices for testing and evaluation. This phase would also encompass human factors engineering to ensure the usability and ergonomic design of the devices, maximizing ease of use for warfighters. Interoperability with other equipment and systems, cybersecurity precautions, as well as considerations for sustainment and logistics, would be critical during the integration process. Insights from previous attempts to integrate EEG into warfighters’ helmets can inform this endeavor (Ko et al., 2019; Von Rosenberg et al., 2016; Zaman et al., 2022). While EEG integration faced challenges related to signal quality, comfort, and practicality, lessons learned from these efforts can guide the development and integration of other neurotechnology devices. Particularly, the importance of user-centered design, robust testing and evaluation, and ongoing lifecycle management to ensure the successful integration and utilization of neuroenhancement technologies in military operations is emphasized.

#### 6.1.7 Developing regulatory and ethical frameworks and guidelines

What are the ethical and regulatory considerations for ES application in military contexts? The use of ES in military contexts for enhancing cognitive functions and performance raises several ethical, legal, safety, and regulatory considerations. These include informed consent, autonomy, maintaining warfighter dignity and morality, potential risks to soldiers’ health and well-being, societal implications, and compliance with existing medical device regulations (Cinel et al., 2019; Davis and Smith, 2019; Puscas, 2020). Military personnel are a potentially vulnerable group who may face pressure to accept neuroenhancement due to authority relationships, hierarchical command structures, and the potential rights they forego when they enlist (Latheef and Henschke, 2020).

Regular and prolonged use of ES may carry yet unknown long-term effects for brain function, mental health, and neurological integrity. While acute effects are often reported as mild (e.g., tingling, itching), the consequences of repeated or long-term use remain largely unknown, raising questions about cumulative risks, dependency, and reversibility. If ES induces lasting neural plasticity or cognitive changes, what are the implications for warfighters once they leave military service? This issue extends to the ethical responsibility of the military and medical oversight bodies in ensuring that enhancement technologies do not compromise the long-term well-being of personnel, during and beyond their service. Another ethical issue is dual-use concerns, specifically the possibility that neuroenhancing technologies developed for military applications could be repurposed for punitive, coercive, or otherwise unauthorized civilian and non-civilian uses. This weaponization of neurotechnology, including potential application in interrogation, must be carefully considered and addressed through policy frameworks and regulatory oversight. An additional concern arises when the enhancement of one function coincides with diminished activity in other brain regions (Cinel et al., 2019). In future multi-domain operations that demand high levels of adaptability and multitasking, this could prove problematic.

For ES to be valuable and justifiable in military contexts, the positive effects must be superior, additive, proliferative, or associated with fewer adverse events than existing enhancement methods such as cognitive training (Davis and Smith, 2019). The use of stimulation to not only restore and maintain, but also aim to enhance human capabilities beyond natural limits raises ethical questions around fairness, merit, and the redefinition of military virtues such as courage, loyalty, and sacrifice (Puscas, 2020). Fairness and equity must also be considered, particularly regarding access to systems and potential performance disparities that can result. If ES confers advantages in certain domains, it could create disparities between enhanced versus non-enhanced personnel, and potentially lead to new stratification within military ranks where some are prioritized for promotions or special roles, whereas others are disadvantaged. Finally, it is possible that ES could create unrealistic expectations of warfighters, or cause overreliance or overestimation of its potential; indeed, if military personnel believe it provides a strong advantage for mitigating stress, fatigue, or other suboptimal states, they may take more risks and jeopardize the safety of themselves and others (Wilde, 1982).

Proactive engagement with stakeholders, including military personnel, ethicists, policymakers, and the broader scientific community, is essential to address these ethical and regulatory challenges, and establish guidelines that balance the potential benefits of ES with ethical and safety considerations in military training and operations. To this aim, a number of frameworks and regulatory guidelines on neuroenhancement are published by various government organizations and academia (Emanuel et al., 2019; Maslen et al., 2014). Furthermore, the Hybrid Minds project seeks to establish a foundational framework for ethically and legally evaluating neurostimulation systems embedded in intelligent neuroprostheses to ensure safety and ethics (Jotterand and Ienca, 2023). This framework is uniquely shaped by insights from user experiences and perspectives, along with input from scientists and engineers, helping to motivate international declarations regarding neurotechnologies and their relevance for human rights (Bublitz, 2024).

## 7 Conclusion

This systematic review highlights the complex effects of ES, including tDCS, tACS, and tVNS, on military-relevant cognitive performance outcomes. While some studies demonstrate potential benefits of ES on tasks like multitasking and visual search, the overall evidence remains mixed, with no significant effects observed on measures of vigilance and inconsistent outcomes across other cognitive domains. Despite the insights provided by the reviewed studies, the generalizability of these findings remains limited due to the relatively small number of studies identified and reviewed. The variability in methodologies, sample sizes, and cognitive tasks assessed further constrains the ability to draw definitive conclusions about the efficacy and safety of ES in military contexts. The scarcity of high-powered, ecologically valid studies highlights the need for more robust investigations that systematically explore ES effects across diverse military tasks and operational conditions. Future research should prioritize larger sample sizes, improved standardization of stimulation parameters, examination of individual differences, and longitudinal studies to better assess the long-term effects and practical viability of ES for performance enhancement in real-world military applications.

Furthermore, the review identified several critical gaps and outstanding questions that must be addressed to advance the application of ES in military settings. These include understanding the generalizability of lab-based findings to real-world military tasks, the frequency and intensity of acute and long-term adverse effects, the impact of repeated ES administration, and the ethical and regulatory considerations surrounding the use of these technologies. The ethical challenges of ES, particularly in military contexts, extend beyond individual safety to broader concerns about informed consent, autonomy, fairness, and the potential for coercion and inequities in hierarchical structures. Additionally, the long-term consequences of ES on neurological integrity and cognitive function remain unclear, necessitating careful oversight to prevent unintended harm to a potentially vulnerable population of end users. Addressing these questions through interdisciplinary collaboration and rigorous research is essential to fully realize the potential of ES for enhancing cognitive performance and operational effectiveness in military personnel while ensuring its responsible and ethical implementation.

## Data Availability

All extracted data are provided in Table 3.

## Author disclaimer

The views expressed in this article are solely those of the authors and do not reflect the official policies or positions of the Department of Army, the Department of Defense, or any other department or agency of the U.S. government. The contents include material subject to © Crown copyright (2025), Dstl. This information is licensed under the Open Government Licence v3.0. To view this licence, visit https://www.nationalarchives.gov.uk/doc/open-government-licence/. Where we have identified any third party copyright information you will need to obtain permission from the copyright holders concerned. Any enquiries regarding this publication should be sent to: centralenquiries@dstl.gov.uk.
