# Supplementary material for: Transcutaneous and transcranial electrical stimulation for enhancing military performance: an update and systematic review
Source: Front Hum Neurosci. 2025 Mar 3;19:1501209. doi: 10.3389/fnhum.2025.1501209 (PMC11911350; doi:10.3389/fnhum.2025.1501209)
Supplement: Supplementary file 1 [file Data_Sheet_1.DOCX]

Supplementary Material

# Example SCOPUS Search Operators

(( AFFIL ( army OR defence OR defense OR navy OR naval OR air AND force OR soldier )) OR ( TITLE-ABS-KEY ( militaries ) OR INDEXTERMS ( "military personnel" ) OR ( TITLE-ABS-KEY ( military ) AND TITLE-ABS-KEY ( personnel ) ) OR TITLE-ABS-KEY ( "military personnel" ) OR TITLE-ABS-KEY ( military ) OR TITLE-ABS-KEY ( "military s" ) OR ( TITLE-ABS-KEY ( defence ) OR TITLE-ABS-KEY ( defense ) ) OR ( INDEXTERMS ( "military personnel" ) OR ( TITLE-ABS-KEY ( military ) AND TITLE-ABS-KEY ( personnel ) ) OR TITLE-ABS-KEY ( "military personnel" ) OR TITLE-ABS-KEY ( soldier ) OR TITLE-ABS-KEY ( soldiers ) OR TITLE-ABS-KEY ( "soldier s" ) ) )AND ( ( TITLE-ABS-KEY ( "transcranial electrical stimulation" ) OR TITLE-ABS-KEY ( "transcranial direct current stimulation" ) OR TITLE-ABS-KEY ( "transcranial alternating current stimulation" ) OR TITLE-ABS-KEY ( "transcranial random noise stimulation" ) OR TITLE-ABS-KEY ( "trigeminal nerve stimulation" ) OR TITLE-ABS-KEY ( "vagus nerve stimulation" ) OR TITLE-ABS-KEY ( "transauricular electrical stimulation" ) OR TITLE-ABS-KEY ( "transcutaneous vagus nerve stimulation" ) OR TITLE-ABS-KEY ( "Transcutaneous auricular vagus nerve stimulation" ) OR TITLE-ABS-KEY ( "cranial electrical stimulation" ) OR TITLE-ABS-KEY ( "trigeminal nerve stimulation" ) OR TITLE-ABS-KEY ( "Transcutaneous Stimulation" ) OR TITLE-ABS-KEY ( transcutaneous AND electrical AND nerve AND stimulation ) OR TITLE-ABS-KEY ( peripheral AND nerve AND stimulation ) ) ) )
